# Prompt Management of Airbag Burn Injuries Leads to Optimized Patient Outcomes: A Pilot Study

**DOI:** 10.7759/cureus.41808

**Published:** 2023-07-13

**Authors:** Grant N Schalet, Stacey L Tannenbaum, Taylor Ouellette, James W Fletcher

**Affiliations:** 1 Department of Surgery, Broward Health Medical Center, Fort Lauderdale, USA; 2 Department of Surgery, Nova Southeastern University Dr. Kiran C. Patel College of Osteopathic Medicine, Fort Lauderdale, USA; 3 Department of Plastic Surgery, Broward Health Medical Center, Fort Lauderdale, USA

**Keywords:** full-thickness burn, partial-thickness burn, dysaesthesia, hyperpigmentation, early treatment, chemical burn, mechanical burn, thermal burn, airbag deployment, airbag burn

## Abstract

Background: Dual airbags are required to be installed and available for use in all motor vehicles since 1997. The National Highway Traffic Safety Administration reported that 50,457 lives were saved by airbags from 1987 to 2017; however, airbag deployment can cause injuries, including thermal and chemical burns, hyperpigmentation, and dysaesthesia. There is little information available in the literature regarding differences in outcomes between promptly visiting a plastic surgeon and waiting for treatment, especially as an injury may not be immediately apparent or patients may not know that airbag burn injuries may be delayed in presenting.

Methods: This is a retrospective cohort pilot study conducted among 14 patients who presented to a plastic surgeon between January 1, 2019 and June 30, 2022 owing to injuries from airbag deployment. An early visit was considered ≤30 days, and a late visit was >30 days. Other variables collected included age, sex, Fitzpatrick skin type, smoking status, comorbidities, type of injury, injury site, pain status, hyper/hypopigmentation, dysaesthesia, epithelialization, and improvements in pain, pigmentation, and dysaesthesia from treatment.

Results: The mean age was 36.0 years (standard deviation (SD) 17.9). The majority were female (85.7%), non-smokers (87.5%), and not diabetic (75.0%). Only six patients (42.9%) visited their doctor within one month of injury. Most patients experienced dysaesthesia (85.7%) and pain (71.4%). Thirteen of the 14 patients had hyperpigmentation or hyperemia, and one had hypopigmentation. Full or slight epithelialization was seen in 35.7%, and nine of the 14 patients had no epithelialization. Ongoing issues were a factor for 64.3% of these patients; 42.9% had ongoing issues with hyperpigmentation. A full recovery was seen in 28.6% of the patients. The patients who saw the plastic surgeon by day 30 or less (early) from the time of injury had a 66.7% improvement in pigmentation and 33.3% resolution in pain. Of those who went to the surgeon beyond 30 days (late), 25% had improvement in pigmentation and 37.5% had resolution of pain. Improvement in dysaesthesia occurred in both groups, but those who saw the plastic surgeon early had 33.3% resolution, while 37.5% of those who went late improved. Of those who went late to the surgeon, only 12.5% had epithelialization, while 66.7% of those who went within 30 days showed signs of (full or slight) epithelialization.

Conclusion: Patients involved in motor vehicle collisions (MVCs) should be informed of the delayed fashion in which airbag burns can develop. An ostensibly mild burn may portend long-term consequences, especially if such injuries are not addressed in a prompt manner. Our study demonstrates how airbag burn injuries and their sequelae are best addressed with early care.

## Introduction

Motor vehicle collisions (MVC) cause more than 7,500 injuries in the United States every day [1]. The National Highway Traffic Safety Administration (NHTSA) estimated that in 2019, 16,629 drivers and 5,586 passengers were injured or killed by MVCs [2]. Since 1997, all new vehicles are required to have dual airbags installed and available for use [1]. Moreover, the NHTSA determined that 50,457 lives were saved by the presence of frontal airbags from 1987 to 2017. Airbags are not intended to inflate for all MVCs [2]; however, in high-velocity MVCs, airbags typically deploy with great speed and force in order to effectively protect occupants. The rapid and explosive nature of this inflation puts individuals at risk for three types of burn injuries - mechanical, thermal, and chemical. Antosia and Partridge (1995) reviewed data collected by the NHTSA and found that most injuries from airbag deployment were to the face (42.0%), wrist (16.8%), forearm (16.3%), and chest (9.6%) [3]. Airbags can also cause skin abrasions and contusions or fractures to the face, fingers, hands or arms, lower legs, and feet, which can happen with passengers who are resting their legs over the glove compartment or instrument panel as the airbag deploys [4].

Mechanical injuries occur from the sheer force of the airbag as it contacts the occupant’s skin. Thermal and chemical burns can occur from an exothermic reaction that produces excessive heat from airbag gases while exposing individuals to caustic alkaline substances. Airbags may cause harm to an out-of-position occupant (e.g., not using a seatbelt) and those too close (within four inches) to the airbag at the time of deployment when the airbag reaches peak force [5]. This situation occurs in shorter drivers or older adult drivers as they tend to sit closer to the steering wheel where the airbag is situated [4]. Airbags are designed to be used in conjunction with a seatbelt restraint. Compliant passengers, i.e., those who wear seat belts regularly, are less likely to fall victim to airbag injuries as often or as severely as non-compliant occupants.

An injury from airbags may not be noticed or prioritized in the emergency department, and patients may be discharged from the hospital only to discover an airbag injury later. The decision to seek timely treatment may have a profound impact on the healing potential of a burn injury. The main purpose of this study is to evaluate whether early (≤30 days) physician intervention and treatment provides patients with proportionally better outcomes in driver and passenger airbag burn injuries compared with late (>30 days) intervention after MVC. The secondary objective of this study is to describe and compare the extent of driver and passenger airbag burn injuries after MVCs.

## Materials and methods

This was a retrospective cohort pilot study conducted at Broward Health Medical Center located in Fort Lauderdale, Florida, USA, and a plastic and reconstructive surgeon's private practice. The sample consisted of drivers who were involved in an MVC where the airbag was deployed; the MVC could have been of any type (e.g., head-on with another vehicle or head-on with an object, T-boned by another vehicle, or sideswiped) and any speed of driving as long as the airbag was released during the collision. Patients 18 years of age and older who were involved in an MVC and developed a burn injury from airbag deployment were included in the study. Airbag deployment could have been from the steering wheel, side airbags, or both. MVC drivers who did not go to the emergency room or a level one trauma center for treatment but presented to the plastic and reconstructive surgery office of this private practitioner were still eligible for this study as long as they reported that the injury they received was likely from an MVC. There were no exclusions in this study.

The outpatient medical records were queried for International Classification of Diseases 10th Revision (ICD-10) codes in order to find eligible patients within the timeframe of January 1, 2019 to June 30, 2022 (*n*=14). Patient records from the electronic medical record (EMR) were only logged retrospectively; that is, none were followed up past June 30, 2022. The EMR was reviewed to collect basic patient demographics and medical history.

Patients were categorized by the time from the injury to the first visit with the plastic surgeon for intervention and treatment of airbag burn injuries, which was recorded in days. An early visit was considered ≤30 days, and a late visit was delineated as >30 days. Other variables collected included age (years); sex (male or female); Fitzpatrick skin type (1-5); smoking status (yes or no); diabetes (yes or no); other comorbidities (yes or no); type of injury (superficial, partial-thickness, or full-thickness burn); injury site (back, neck, nose, orbital region, mouth, upper extremity, hand, face, ear, or lower extremity); pain status (yes or no); hyper/hypopigmentation (yes, no, slight, or hypopigmentation); dysaesthesia (yes or no); epithelialization (yes, slight, or no); improvements in pain, pigmentation, and dysaesthesia (no improvement, slight improvement, or resolved for each); ongoing issues (hyperpigmentation, none, or others), and treatment.

Demographic data are displayed by means and standard deviation (SD) for numeric data (range, median, and interquartile range) and were only reported for length of time from injury to presenting at the plastic surgeon. Frequencies and percentages were presented for categorical data. All data are presented as proportions of the total sample and displayed by early and late visit to the plastic surgeon for comparison. However, due to the small sample size and because this was a pilot study, no inferential statistics were performed on these data. All demographic analyses were performed using IBM SPSS Statistics for Windows, Version 28 (Released 2021; IBM Corp., Armonk, New York, United States) [6].

The Institutional Review Board (IRB) approved this study as having minimal risk and provided an exempt status; IRB rules dictate that informed consent is not required for this retrospective study.

## Results

**Figure 1:**
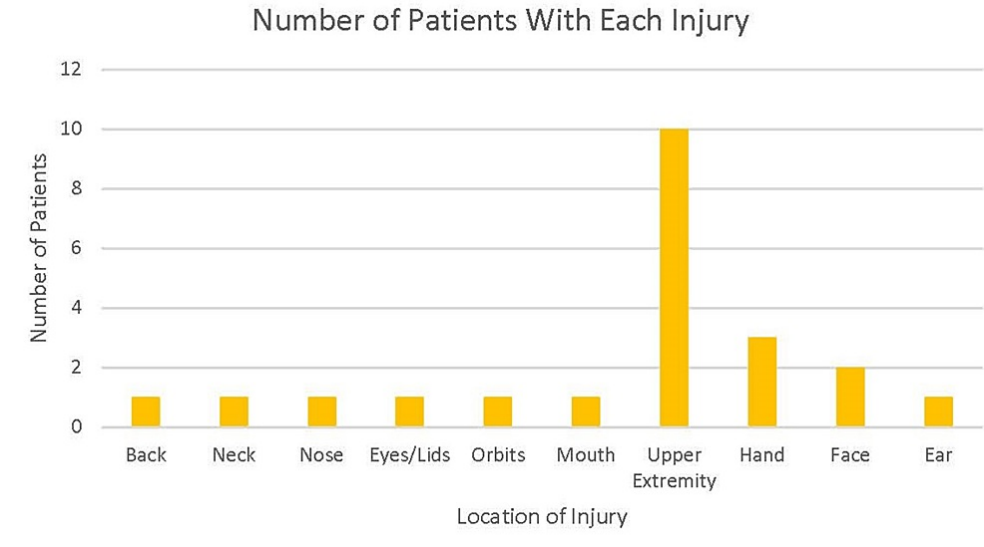
Number of injuries from airbag deployment of each body area affected (n=14)

The age, sex, location of burn, and time till first visit of each patient are depicted in Table 1. The mean age of the sample was 36.0 years with an SD of 17.9. The majority of the sample was female (85.7%), were non-smokers (87.5%), and not diabetic (75.0%). The most common comorbidity was hypertension (36.4%) or had no comorbidities (42.9%), and the most common Fitzpatrick skin type was two or three (42.8%). Only six patients (42.9%) visited their doctor within one month of injury (Table 1). The range in length of time from injury to outpatient visit was 3-134 days, with the median number of days being 44 and the interquartile range being 58.3. Injury to the upper extremity was the most common body site injury (71.4%), followed by injury to the hand (21.4%) (Figure 1).

**Table 1:** Characteristics of airbag injury patients who presented to one private plastic and reconstructive surgery practice office from January 1, 2019 to June 30, 2022

Patient	Age	Sex	Location of Burn	Time Till First Visit (days)
1	33	Male	Hand	35
2	20	Female	Nose, orbital,	9
3	45	Male	Upper extremity	103
4	27	Female	Upper extremity	3
5	33	Female	Upper extremity	53
6	22	Female	Upper extremity	134
7	66	Female	Upper extremity	81
8	50	Female	Upper extremity, hand	53
9	29	Female	Upper extremity	17
10	44	Female	Back	11
11	75	Female	Upper extremity, hand	22
12	26	Female	Upper extremity, mouth, face, ear	59
13	18	Female	Nose	110
14	16	Female	Upper extremity, face	18

 

**Table 2:** Clinical sequelae of airbag burn injuries of patients from one private plastic and reconstructive practice office from January 1, 2019 to June 30, 2022 1st degree: superficial burn; 2nd degree: partial-thickness burn; 3rd degree: full-thickness burn *missing data

Patient	Degree of burn (1st, 2nd, 3rd)	Epithelialization (Yes or No)	Dysaesthesia (Yes or No)	Pain (Yes or No)	Hyperpigmentation (Yes or No)	Time till first visit (days)
1	1st	No	Yes	No	Yes	35
2	1st	No	Yes	Yes	Yes	9
3	3rd	No	Yes	Yes	Yes	103
4	2nd	Yes	No	Yes	Yes	3
5	2nd	No	Yes	Yes	Yes	53
6	2nd	No	Yes	No	Hypopigmentation	134
7	2nd	Yes	Yes	Yes	Yes	81
8	3rd	No	Yes	Yes	Yes	53
9	2nd	Yes	Yes	Yes	Yes	17
10	2nd	Yes	Yes	Yes	Yes	11
11	2nd and 3rd	Yes	*	*	Yes	22
12	1st	No	Yes	No	Yes	59
13	2nd	No	Yes	Yes	Yes	110
14	2nd and 3rd	No	Yes	Yes	Yes	18

The clinical sequelae of airbag burn injuries in the sample are displayed in Table 2. Partial-thickness skin loss (second-degree burn) occurred in 64.3%, while full-thickness skin loss (third-degree burn) was evident in 28.6%. Most patients experienced dysaesthesia (85.7%) and pain (71.4%), while 13 out of the 14 patients had hyperpigmentation or hyperemia and one had hypopigmentation. Full or slight epithelialization was seen in 35.7% of the patients, while nine of the 14 patients had no epithelialization. Ongoing symptoms were a factor for 64.3% of these patients; 42.9% had ongoing issues with hyperpigmentation. Other issues included hypertrophic scarring, fat atrophy, and ongoing pain to palpation. A full recovery was seen in 28.6% of the patients (Table 3). Patients who saw the plastic and reconstructive surgeon early, or within 30 days or less, had 66.7% improvement in pigmentation and 33.3% resolution in pain issues, although there were many missing values for improvement in hyperpigmentation (35.7%) and pain (50%). In the late presentation group or those who went to the surgeon beyond 30 days, 25.0% had improved pigmentation, and 37.5% had improvement in pain. Improvement in dysaesthesia occurred in both groups, but those who saw the plastic surgeon early had 33.3% resolution, while 37.5% of those who went late demonstrated improvement. However, all had residual symptoms. Of those who went late to the surgeon, only 12.5% had epithelialization, while 66.7% of those who went early showed signs of (full or slight) epithelialization. Treatments varied from sun protection factor (SPF) usage and avoidance of ultraviolet (UV) light to topical ointments, including cortisone, Silvadene cream, Neosporin, or bacitracin, silicone sheet, compression, platelet-rich plasma (PRP) injections, and, in cases of more severe burns, fat grafting (Table 3).

**Table 3:** Final clinical outcomes and ongoing symptoms of airbag burn injury patients by time till the first visit to the surgeon (early versus late) from one private plastic and reconstructive surgery office from January 1, 2019 to June 30, 2022 Early: 30 days or less before seeing a plastic surgeon; late: more than 30 days before seeing a plastic surgeon; PRP: platelet-rich plasma; SPF: sun protection factor; UV: ultraviolet

Outcomes	Total n (%)	Early n (%)	Late n (%)
Improvement in dysaesthesia	5 (35.7)	2 (33.3)	3 (37.5)
Improvement in hyperpigmentation	6 (42.9)	4 (66.7)	2 (25.0)
Improvement in pain	5 (35.7)	2 (33.3)	3 (37.5)
Ongoing issues	9 (64.3)	4 (66.7)	5 (62.5)
Full recovery	4 (28.6)	2 (33.3)	2 (25.0)
Treatments			
Observe	4 (28.6)	1 (16.7)	3 (37.5)
SPF/UV avoidance	4 (28.6)	3 (50.0)	1 (12.5)
Silvadene, Neosporin, bacitracin, or cortisone	5 (35.7)	3 (50.0)	2 (33.3)
PRP injection	1 (7.1)	0	1 (12.5)
Fat grafting	1 (7.1)	0	1 (12.5)
Debridement	1 (7.1)	0	1 (12.5)
Silicone sheet	1 (7.1)	1 (16.7)	0
Compression	1 (7.1)	1 (16.7)	0
Vitamin E	1 (7.1)	1 (16.7)	0
None	1 (7.1)	0	1 (12.5)

## Discussion

To the best of our knowledge, this is the first study that evaluated injuries from airbag deployment while assessing whether the timing of patient presentation influenced clinical outcomes. The impetus of this study was the increase in airbag-induced burn patients presenting to the office of a private practice plastic surgeon.

Airbags have become an essential part of motor vehicle passenger safety. A study reported a 28% decrease in fatalities when used in combination with a seatbelt [7]. However, the incidence of airbag injuries - including burns - has increased over the years, with burns comprising 8% of these injuries [7].

During an MVC, airbags located in the front of the automobile deploy through a series of key events. First, sensors inside the vehicle receive an electrical signal of substantial deceleration as the vehicle moves forward. The airbag device envelopes a cartridge containing about 70 grams of compressed sodium azide gas or propellant [8,9]. During sudden deceleration, sensors activate the cartridge encompassing this material. Once the sodium azide ignites, a chain reaction proceeds where a combined release of nitrogen, carbon dioxide, carbon monoxide, ammonia, and alkaline aerosol occurs [1,7]. Metallic oxides and sodium hydroxide gases are produced and partly released through the airbag vents into the passenger compartment [8,9]. Then, in a period of approximately 100 milliseconds, the airbag instantaneously deploys in an exothermic reaction that generates heat upwards of 500℃ [7]. Airbags have vents that gradually release the gas after inflation as the driver or passenger encounters the bag.

Thermal burn injuries arise as a result of either direct or indirect causes. Direct burn injuries are often both mechanical and thermal in nature. The direct contact of the airbag with skin or clothing, in combination with the high temperature released from the bag’s inflation, can lead to cutaneous injuries. Long-term sequelae, including hyperpigmentation, pain, and dysaesthesia, are possible, although as seen in our study, some patients fully recover from these injuries. According to the literature, superficial thermal burns have the appearance of a cigarette burn with sporadic blisters [7,10]. During the venting phase, thermal burns can result secondary to air contact with the skin. The prognosis of thermal burn injuries is better when appropriately treated, typically with a topical antibiotic ointment and local wound management, ideally early in the recovery process. In this study, patients were separated by early versus late presentation to the plastic surgeon and compared for outcomes. Due to the small sample size and some missing data, demonstration of statistically significant differences from time to presentation was not possible. In this study, patients who presented to the plastic surgeon within 30 days of the MVC experienced complete recovery proportionally more than those patients who presented in a delayed fashion. As noted in the results, of patients who presented late, 25.0% had improved pigmentation, 37.5% had improvement in pain, but none had pain resolution. However, among the patients who presented to the surgeon’s office promptly (30 days or less), 66.7% had improvement in pigmentation and 33.3% had improvement in pain.

The more sinister mechanism of injury - chemical burns - can produce lasting harm that goes unnoticed in the initial period after the MVC. Alkaline chemicals, including aerosolized sodium hydroxide and sodium carbonate, along with other chemicals created during airbag inflation, can make contact with organic fluids, including sweat and tears [10-12]. On the biochemical level, alkalines saponify fatty acids and derange mucopolysaccharides [13]. Protein degradation and inflammatory vasodilation follow, setting the stage for bacterial infections. The treatment modalities for chemical burns vary, but oftentimes, debridement, silver sulfadiazine, topical antibiotics, and/or topical steroids are used. In contrast to thermal burn injuries, alkaline chemicals can cause burns of greater depth and with a diminished likelihood of returning to baseline after healing.

Three parties can play a role in reducing the risk of burn injuries: surgeons, airbag manufacturers, and drivers. As noted by Goshev et al. (2017), airbag-induced burn injuries may either be an unavoidable downside of the protective mechanism of the airbag or rather secondary to infrequent manufacturing defects [8]. Heimbach (2000) noted that the stronger alkaline gases are no longer produced in newer model cars, resulting in fewer chemical burns [14]. From the perspective of surgeons, prompt and careful evaluation of burn injuries is vital to positive long-term outcomes. Surgeons should consider proactively treating even superficial burns, as these may be a harbinger of more extensive underlying chemical damage. A meticulous history and physical examination are essential. When it comes to suspecting a chemical injury, it would be prudent to obtain the pH of the wound at the time of patient presentation in order to most appropriately tailor treatment [15].

The airbag itself should be kept clear of any conductive materials, thus helping to minimize the risk of unnecessary heat transfer during deployment, as proposed by Mercer and Sidhu [16]. Vents should be made larger to minimize the risk of thermal injury during deflation [16], and airbags should be turned off in low-velocity city traffic (less than 60 kilometers per hour or approximately 35-40 miles per hour) and only activated at higher speeds [17]. While this may be beneficial in theory, the authors of this paper believe that, prior to making this adjustment, future research should further evaluate low-velocity burn injuries as airbags may still offer a vital protective mechanism, even in the context of lower-speed collisions.

Our study had some limitations. Our sample size was small and contained missing data points. The authors of this study feel that with a larger sample size, there would be statistically significant differences in patient outcomes based on time to treatment. The patients were selected from a high-volume private practice of a plastic and reconstructive surgeon in Florida. Relative to the principal investigator’s overall patient base, airbag-induced burns are a small portion, so this study’s design was a cohort pilot study. Nevertheless, by comparing our findings to the literature and using a 30-day cut point, we demonstrated some improvement in several burn sequelae when prompt management of burn injuries was accomplished in the outpatient setting. There was an inherent selection bias within this study. That is, only those patients with a burn injury that was recognized as emerging from the MVC presented to the office. It is conceivable that more patients with airbag burn injuries chose not to visit a plastic surgeon but rather their primary care physician, or perhaps the injury was not noticed promptly, connected with the MVC, or discovered at all; chemical burn injuries in particular may form in an insidious fashion and lead the patient to delayed patient-physician visits. Alternatively, the strength of this study is that it provides novel information regarding the importance of prompt evaluation and treatment of airbag burn injuries. Most of the literature address the manner in which these injuries are treated but fail to appropriately discuss the significance of seeing these patients in the office soon after the MVC.

## Conclusions

After an MVC, injuries occurring from rapid airbag deployment may not be apparent, high priority, or even discussed upon initial treatment in a trauma center or emergency department. Patients involved in MVCs should be educated about the possible delayed manner in which wounds can develop after airbag deployment. Promptly attending to airbag burn injuries is beneficial to not only addressing initial symptoms but also minimizing the risk of permanent cosmetic and functional damage from these types of injuries.
